# A novel strategy for bioactive natural products targeting NLRP3 inflammasome in Alzheimer’s disease

**DOI:** 10.3389/fphar.2022.1077222

**Published:** 2023-01-09

**Authors:** Zhiyou Yang, Junxin Liu, Shuai Wei, Jiahang Deng, Xinyue Feng, Shucheng Liu, Mingxin Liu

**Affiliations:** ^1^ Guangdong Provincial Key Laboratory of Aquatic Product Processing and Safety, Guangdong Province Engineering Laboratory for Marine Biological Products, Guangdong Provincial Engineering Technology Research Center of Seafood, Key Laboratory of Advanced Processing of Aquatic Product of Guangdong Higher Education Institution, College of Food Science and Technology, Guangdong Ocean University, Zhanjiang, China; ^2^ Collaborative Innovation Centre of Seafood Deep Processing, Dalian Polytechnic University, Dalian, China; ^3^ College of Electrical and Information Engineering, Guangdong Ocean University, Zhanjiang, China

**Keywords:** NLRP3 inflammasome, Alzheimer’s disease, natural products, microglia polarization, neuroinflammation

## Abstract

Alzheimer’s disease (AD), the most common type of dementia, is an ageing-related progressive neurodegenerative brain disorder. Extracellular neuritic plaques composed of misfolded amyloid β (Aβ) proteins and intracellular neurofibrillary tangles formed by hyperphosphorylated tau protein are the two classical characteristics of AD. Aβ and tau pathologies induce neurite atrophy and neuronal apoptosis, leading to cognitive, language, and behavioral deficits. For decades, researchers have made great efforts to explore the pathogens and therapeutics of AD; however, its intrinsic mechanism remains unclear and there are still no well-established strategies to restore or even prevent this disease. Therefore, it would be beneficial for the establishment of novel therapeutic strategy to determine the intrinsic molecular mechanism that is interrelated with the initiation and progression of AD. A variety of evidence indicates that neuroinflammation plays a crucial role in the pathogenesis of AD. Nucleotide-binding oligomerization domain (NOD)-like receptor pyrin domain-containing protein 3 (NLRP3) is a key inflammasome sensor of cellular stress and infection that is involved in the innate immune system. In response to a wide range of stimuli like Aβ, NLRP3 assembles apoptosis-associated speck-like protein (ASC) and procaspase-1 into an inflammasome complex to induce the caspase-1 mediated secretion of interleukin (IL)-1β/IL-18 in M1 polarized microglia, triggering the pathophysiological changes and cognitive decline of AD. Therefore, targeting NLRP3 inflammasome seems an efficient path for AD treatment *via* regulating brain immune microenvironment. Furthermore, accumulating evidence indicates that traditional Chinese medicine (TCM) exerts beneficial effects on AD *via* NLRP3 inflammasome inactivation. In this review, we summarize current reports on the role and activated mechanisms of the NLRP3 inflammasome in the pathogenesis of AD. We also review the natural products for attenuating neuroinflammation by targeting NLRP3 inflammasome activation, which provides useful clues for developing novel AD treatments.

## 1 Introduction

Approximately 50 million people worldwide live with dementia, and this is projected to increase to 152 million by 2050. Alzheimer’s disease (AD) is a progressive neurodegenerative disease which accounts for 60%–70% of dementia. Currently, China has about 9.5 million AD patients, the highest in the world (Livingston et al., 2020). Extracellular neuritic Aβ plaques and intracellular neurofibrillary tangles (NFTs) in the brain are two pathological hallmarks of AD. The clinical characteristics of AD patients include memory and cognitive dysfunction, and language and behavioral disorders (Feng et al., 2020). Neuritic plaques comprise misfolded Aβ fibril proteins generated from Aβ precursor proteins (APP) by β- and γ-secretase, while NFTs are accumulated and deposited by hyperphosphorylated tau protein. Although several hypotheses—including the amyloid hypothesis, the tau hypothesis, inflammation hypothesis, microglial glucose metabolism disruption hypothesis, and autoimmune disorder hypothesis—have been proposed to explain the pathogenesis of AD, the detailed mechanism underlying neuronal loss and cognitive deficits in AD remains elusive.

Recently, growing evidence has supported a critical role for immune regulation in the progression or even initiation of AD. APP-α or Aβ fibrils trigger the activation of microglia and enhance their production of neurotoxins, such as inducible nitric oxide synthase (iNOS) and interleukin (IL)-1β (Barger and Harmon, 1997). The secreted IL-1α, tumor necrosis factor (TNF), and complement component 1q (C1q) from activated microglia induce the polarization of A1 astrocytes, which contribute to the death of neurons and oligodendrocytes in AD (Liddelow et al., 2017). IL-1β, one of the main mediators of innate immune response, is elevated in the brain of AD patients and can be associated with the progression and early onset of AD. Inflammasomes are involved in initiating and sustaining the innate immune response in the peripheral and central nervous system. NLRP3 is highly expressed in microglia, and it is intercorrelated with an assortment of chronic inflammatory diseases for sensing danger-associated molecular patterns and aggregated proteins including Aβ (Feng et al., 2020). Once microglia are stimulated by Aβ, NLRP3 recruits adaptor protein ASC and procaspase-1 into an inflammasome complex, which is important for the cleavage of procaspase-1 into an inflammasome effector protein caspase-1 *via* autocatalysis. Subsequently, the proinflammatory cytokines—especially pro-IL-18 and pro-IL-1β—will be cleaved by caspase-1 into mature forms of IL-18 and IL-1β, eventually resulting in immune responses, neuronal death, and pyroptosis (Liang et al., 2022). In APP/(presenilin-1) PS1 transgenic mice, NLRP3 inflammasome activation mediates microglia to exhibit an inflammatory M1 phenotype, which exerts a low expression of degradation enzymes and is unable to engulf and degrade Aβ, resulting in increased Aβ deposits (Yang et al., 2019a). Knockout of microglial NLRP3 or caspase-1 polarize microglia into M2 phenotype is accompanied by enhanced Aβ clearance and improved learning and cognitive memory function (Heneka et al., 2013). These results indicate that suppressing NLRP3 inflammasome activation may reduce neuroinflammation and ameliorate the pathophysiological processes of AD, and may therefore be a novel therapeutic strategy for AD. Although the mechanisms of NLRP3 inflammasome activation have been reviewed (Zhang et al., 2020a; Zhang et al., 2020b), the mechanism of inflammasome activation has not been fully understood, as well as the relationship between microglia and AD pathologies.

Generally speaking, natural medicine-derived therapeutic agents are superior to structural modified synthetic drugs for their higher safety and fewer side effects. Current evidence indicates that natural products and their bioactive molecules are promising potential drug leads for the treatment of AD *via* inhibiting NLRP3 inflammasome mediated neuroinflammation (Bagherniya et al., 2021). Some reviews have summarized medicinal plants and natural products as NLRP3 inhibitors (Bagherniya et al., 2021) and natural inhibitors targeting AD and Parkinson’s disease (Lee et al., 2021); however, the inhibitors, including formulas, extracts, and single molecules, were not fully reviewed. Therefore, we here summarize the activation mechanism of the NLRP3 inflammasome and its pathological role in AD by searching for the words “inflammasome” and “Alzheimer’s disease” in databases including Web of Science, PubMed, Google Scholar, Sci-hub, and SciFinder. In addition, we attempt to update the current knowledge of the existed natural products that target NLRP3 inflammasome for AD by searching for “inflammasome”, “Alzheimer’s disease”, and “compound/natural products/formula/extracts”. The references collected range from 1989 to 2022.

## 2 The activating of NLRP3 inflammasome in microglia of AD

The NLRP3 inflammasome mediates the activation and secretion of pro-inflammatory cytokines by immune cells, especially microglia in the brain (Hanslik and Ulland, 2020). Initiating and activating signals are required to activate NLRP3 inflammasome. Damage-associated molecular patterns (DAMPs) or pathogen-associated molecular patterns (PAMPs), the classical initiation signals, induce the transcription of pro-IL-1β, pro-IL-18, and NLRP3 *via* TLR/IL-1R/MyD88 dependent NF-κB activation (Yang et al., 2020). Subsequently, various activating signals, including reactive oxygen species (ROS), efflux of potassium or chloride ions, and lysosomal damage, promote the formation of NLRP3 inflammasome, including sensor protein NLRP3 and apoptosis-associated speck-like protein (ASC) containing a caspase recruitment domain. Assembled ASC forms a multimeric complex, commonly called “speck,” which activates procaspase-1 to cleave pro-IL-1β and pro-IL-18 proteins into their mature forms (Swanson et al., 2019).

**FIGURE 1:**
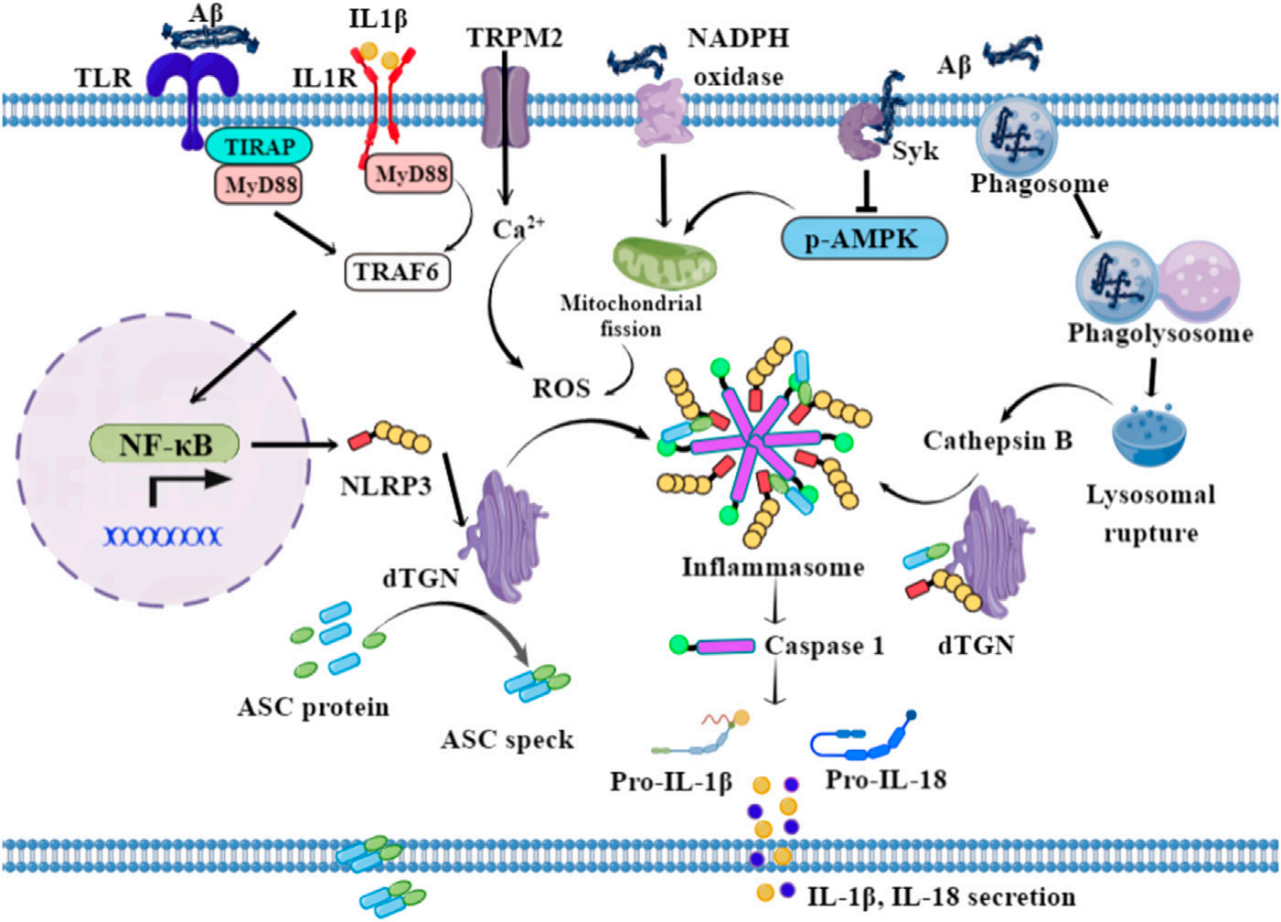
Possible mechanisms of NLRP3 activation in microglia. In the initiation signal, Aβ fibrils bind to TLR, and drive the transcription of NLRP3, pro-IL-1β, and pro-IL-18 *via* NF-κB signaling. In the activating signal, the lysosomal-rupture pathway and ROS generation pathway are the two classical routes for NLRP3-mediated caspase-1 activation. The phagocytosis of aggregated Aβ by microglia triggers lysosomal rupture and the subsequent release of cathepsin B into the cytosol. In addition, Aβ triggers Ca2^+^ influx *via* TRPM2 and activates NADPH oxidase and Syk, subsequently inducing mitochondrial fragmentation and the generation of ROS. Therefore, the stimuli—including ROS and cathepsin B—drive the dispersed transGolgi network (dTGN) to recruit NLRP3 through ionic bonding, leading to adaptor protein ASC polymerization and downstream signaling activation.

In AD, small Aβ oligomers and protofibrils directly interact with NLRP3 and promote NLRP3 and ASC interaction in a cell free system (Nakanishi et al., 2020) and induce NLRP3 inflammasome activation in primary microglia (Lučiūnaitė et al., 2020). In the initiation signals of Aβ-induced NLRP3 activation, aggregated Aβ binds to TLR4 and forms complexes, triggering TLR/MyD88 dependent activation of NF-κB and promoting the translocation of NF-κB from the cytoplasm to the nucleus. The transcription of pro-interleukin (IL)-1β, pro-IL-18, and NLRP3 are subsequently initiated (Figure 1). MyD88-deficiency decreases microglial activation and cerebral Aβ deposits and improves spatial learning in APPswe/PS1dE9 mice (Lim et al., 2012). MyD88 deficiency consistently enhances Aβ peptide phagocytosis by microglia/macrophages and inflammatory activation *in vitro*, microglial replenishment with MyD88 deficient bone marrow cells, improves cognitive functions by enhancing Aβ phagocytosis, and reduces inflammatory activation in AD mouse models—including APP/PS1 and TgCRND8 mice (Hao et al., 2011). Furthermore, the secreted IL-1β binds to IL-1R and accelerates NLRP3 transcription through IL-1R/MyD88/NF-κB signaling (Zhang et al., 2020b). TRAF6 deficiency specifically inhibits TLR/IL-1R priming-initiated caspase-1 cleavage, pyroptosis, and the secretion of presynthesized IL-18, indicating the critical role of TRAF6 in the formation of NLRP3 inflammasome (Xing et al., 2017). Collectively, the findings suggest that TLR4/IL-1R-MyD88-TRAF6 signaling is involved in the initiation signals of NLRP3 inflammasome activation in AD mouse models (Figure 1).

In the activating signal, the lysosomal rupture pathway and the ROS generation pathway are the two classical routes for NLRP3-mediated caspase-1 activation in AD (Figure 1). When aggregated Aβ is phagocytosed by microglia, phagosome combines with lysosome, leading to lysosomal rupture and the subsequent release of cathepsin B into the cytosol. The lysosomal rupture alone can be an endogenous signal for NLRP3 activation, thus stimulating caspase-1 maturation and IL-1β/18, as well as ASC speck secretion (Halle et al., 2008). ASC specks bind to Aβ and seed the surrounding parenchyma, leading to further Aβ aggregation. Aggregated Aβ in turn bind to TLR and induce the activation of the MyD88 pathway. ROS production is another way to activate NLRP3 and caspase-1 and is dependent on mitochondrial function (Zhou et al., 2011). Aβ oligomer-induced IL-1β secretion is dose-dependently decreased by the ROS scavenger N-acetylcysteine, while an NADPH oxidase-specific inhibitor, gp91ds-tat, also dose-dependently decreased Aβ oligomer-induced IL-1β secretion as well as caspase-1 activity, indicating that Aβ oligomer-induced IL-1β secretion is partially dependent on NADPH oxidase (Parajuli et al., 2013). Meanwhile, Aβ42 activates spleen tyrosine kinase (Syk), leading to the inhibition of AMPK phosphorylation in mouse primary microglia. This signaling induces mitochondrial fragmentation and generation of ROS, thereby activating the NLRP3 inflammasome. The inhibition of spleen tyrosine kinase (Syk) and activation of AMPK protects Aβ42-induced mitochondrial hyperfission and inflammasome activation in LPS-primed microglia (Jung et al., 2022). In addition, high expression of ROS activates the transient receptor-potential melastatin 2 (TRPM2) channels, causing intracellular calcium increment; TRPM2 deficiency inhibits caspase-1 activation in microglial cells, signifying the role of TRPM2 channel in ROS-induced NLRP3 activation after exposure to Aβ (Aminzadeh et al., 2018). Furthermore, Aβ exposure triggers microglia metabolic reprogramming from oxidative phosphorylation to glycolysis in mTOR-HIF-1α-dependent signaling, ultimately leading to the decreased production of lactate and a disrupted NAD^+^/NADH ratio, promoting mitochondrial ROS production and NLRP3 inflammasome activation (Baik et al., 2019; Hughes and O'Neill, 2018). How, then, do these stimuli activate NLRP3? Golgi apparatus has a critical role in this process. NLRP3 is recruited to the dispersed transGolgi network (dTGN) through ionic bonding between its conserved polybasic region and negatively charged phosphatidylinositol-4-phosphate (PtdIns4P) on the dTGN. NLRP3 is then aggregated into multiple puncta by the scaffold effects of dTGN, leading to polymerization of the adaptor protein ASC, thereby activating the downstream signaling (Chen J and Chen ZJ, 2018).

The final activation of NLRP3 inflammasome promotes the cleavage of procaspase-1 into activated caspase-1, and the subsequent secretion of inflammatory factors IL-1β, IL-18, as well as of ASC specks in AD. Taken together, the mechanisms of Aβ-induced activation of NLRP3 inflammasome might be more complex, thus requiring this detailed and precise mechanism need to be investigated to clarify the crosstalk between NLRP3 activation and other signaling pathways in AD.

## 3 The role of NLRP3 inflammasome in the initiation and progression of AD

Microglia can be divided into the classical M1 phenotype and the alternative M2 phenotype (Tang and Le, 2016). M1 microglia express iNOS and CD16/32 marker proteins, while M2 express CD206, Arginase I, and Ym1 (Yang et al., 2019a). M1 microglia secrete pro-inflammatory cytokines such as IL-1β and IL-18, which promote neuroinflammation and neuronal apoptosis. M2 microglia polarization contributes to the release of anti-inflammatory cytokines such as IL-4 and IL-10, resulting in anti-neuroinflammation, neurite regeneration, and oligodendrogenesis. Aβ is a representative M1 microglial stimuli (Yang et al., 2019b). In animal models of AD, activated M1 microglia (release of IL-1β and TNF-α) are significantly increased, while M2 microglia (release of IGF-1 and IL-10) are decreased (Wei and Li, 2022); a similar situation is observed in human AD brains (Walker and Lue, 2015). Furthermore, Aβ degradation enzymes, neprilysin, and insulin degradation enzyme are downregulated in iNOS^+^CD206^-^ M1 microglia and upregulated in iNOS^−^CD206^+^ M2 microglia after treatment of naringenin to primary mouse cortical microglia, indicating that M2 microglia is important for phagocytosis and the degradation of Aβ plaques (Yang et al., 2019a).

TLR4 is an initial signal for the transcription of NLRP3 and pro-IL-1β after binding with Aβ. TLR4 inhibition provides neuroprotection and promotes a microglial switch from the inflammatory M1 phenotype to the protective M2 phenotype in APP/PS1 transgenic AD mice, accompanied by a reduction of MyD88, NF-κB, and NLRP3 (Cui et al., 2020). Aβ fibrils promote IL-1β release in an NLRP3- and ASC-dependent manner in cultured microglia (Halle et al., 2008). Reports have also shown that caspase-1 and IL-1β are elevated in activated microglia from the brains of AD animals and patients (Griffin et al., 1989; Heneka et al., 2013). Knockout of NLRP3 leads to decreased Aβ levels and the deposition and amelioration of memory deficits in APP/PS1/NLRP3^−/−^ mice. The M2 microglial phenotype is significantly increased in NLRP3 knockout mice, and thus promotes the phagocytosis and clearance of Aβ plaques (Heneka et al., 2013). NLRP3 inflammasome inhibitor Mcc950 ameliorates synaptic plasticity deficits in a McGill-R-Thy1-APP rat model of AD, indicating the damaging effects of NLRP3 inflammasome in synaptic dysfunction (Qi et al., 2018). Excessive NLRP3 activation and elevated IL-1β levels in microglia also promote tau hyperphosphorylation, neurofibrillary tangles, and synaptic dysfunction in AD by inducing a detrimental chronic inflammatory reaction (Sheng et al., 2000; Heneka, 2017). Knockout of NLRP3 consistently reduces levels of tau hyperphosphorylation in the hippocampus and rescues the spatial memory deficits present in Tau22 mice. Meanwhile, the activity of tau phosphorylation-regulated kinases such as CaMKII-α was inhibited and that of PP2A was promoted in Tau22/Nlrp3^−/−^ mice. Tau hyperphosphorylation in the CA1 region was highly induced in APP/PS1 brain homogenate-injected Tau22 but not Tau22/Nlrp3^−/−^ mice (Ising et al., 2019). Thus, microglia and NLRP3 inflammasome activation play a key role in tau pathologies and are involved in the beta amyloid-cascade initiation of AD (Zhang et al., 2020a).

The intrahippocampal injection of ASC specks in APP/PS1 mice induces the spread of Aβ deposits, while ASC deficiency inhibits the spread of Aβ and ameliorated cognitive deficits in APP/PS1 mice (Venegas et al., 2017). Thus, a release of ASC specks from pyroptotic microglia occurred after NLRP3 inflammasome activation; these are packed with Aβ in prion-like seeding and aggregate the activated microglia, leading to AD progression. In early AD patients, ASC-bound Aβ is found in the brain, and levels of IL-1β and caspase-1 activity are significantly increased, supporting the above hypothesis (Venegas et al., 2017). In addition, Tau aggregates also acting as prion-like Tau seeds can activate NLRP3-ASC inflammasome, while ASC deficiency decreases non-exogenously seeded Tau pathology in Tau transgenic mice (Stancu et al., 2019).

**FIGURE 2:**
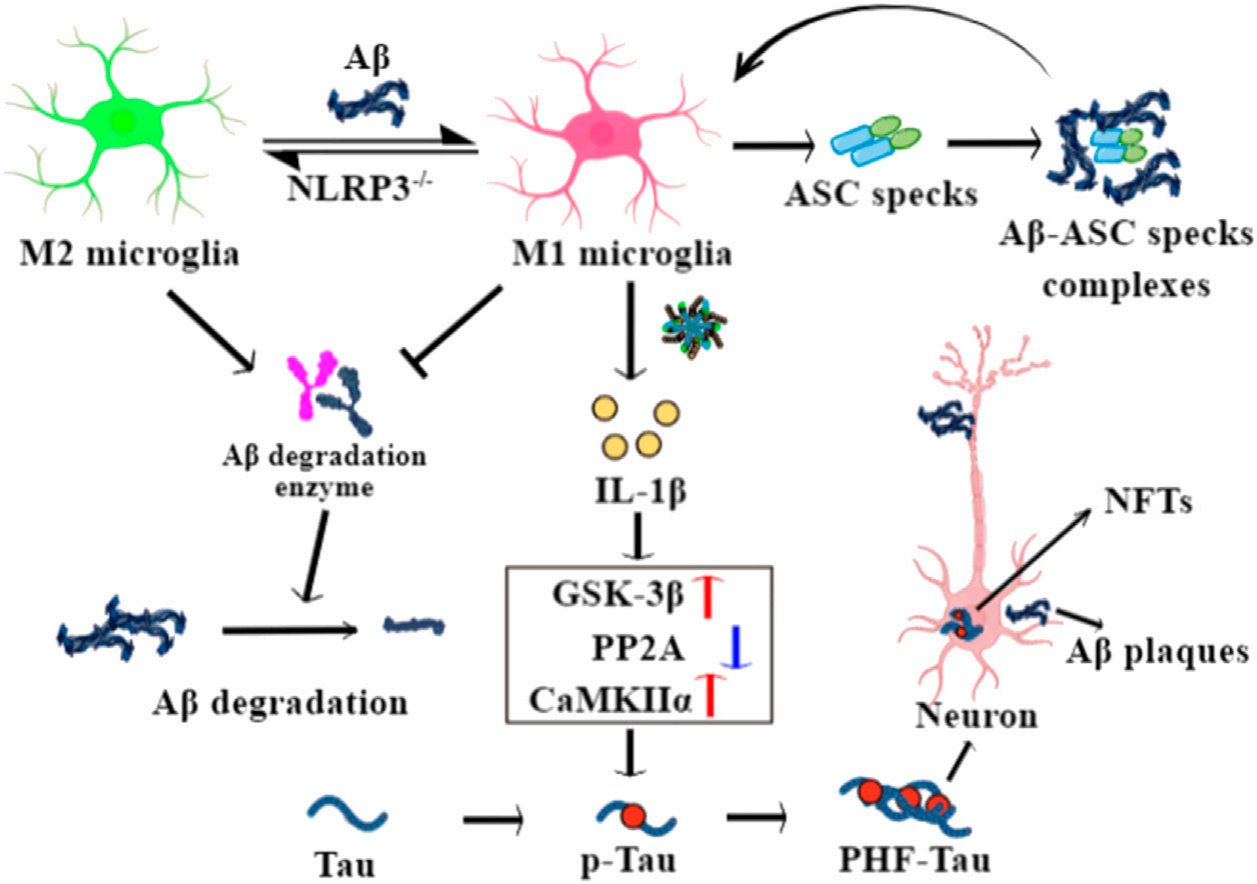
The role of NLRP3 inflammasome in the pathophysiological processes of Alzheimer’s disease (AD). Aβ drives a microglial switch from the protective M2 to the inflammatory M1 phenotypes after binding with TLR4, while NLRP3 knockout reverses it. M1 microglia show decreased phagocytosis ability and low expression of Aβ degradation enzymes, leading to Aβ deposits. Meanwhile, secreted ASC specks from pyroptotic microglia bind to Aβ and seed the surrounding cells to further Aβ aggregation. IL-1β released from M1 microglia enhances the activity of GSK-3β and CaMKIIα enzymes and inhibits PP2A activity in neurons, with phosphorylate tau forming paired helical filament (PHF)-tau and neuronal fibrillary tangles.

These studies shed further light on the possible functional mechanisms of the activated NLRP3 inflammasome in the pathogenesis and progression of AD (Figure 2).

## 4 Natural products as potential inflammasome inhibitors for the treatment of AD

Microglial NLRP3 inflammasome activation is a crucial node that probably promotes or even initiates the pathogenesis of AD. Reports have shown that NLRP3 or caspase-1 knockout remarkably increases Aβ clearance and alleviates cognitive impairment in an AD mouse model (Heneka et al., 2013). This suggests that inflammasome activation plays an important role in the development of cognitive dysfunctions and pathological changes in AD mice. Thus, the intervention of NLRP3 inflammasome activation at the molecular level may be a novel therapy for AD. In the following section, we review and summarize in detail the role of natural medicine-derived therapeutic NLRP3 inflammasome inhibitors in the treatment of AD (Table 1).

**TABLE 1:** Formula, medicinal mixture, extracts, and natural occurring compounds for AD treatment by targeting NLRP3 inflammasome.

Name	Composition/Type	Cell or animal model	Administered method	Main outcome	Ref.
Formula or medicinal mixture					
MBN	—	5×FAD mice	40 mg/kg/day, p.o. for 16 weeks	Inhibition of procaspase-1	Kim et al. (2022)
				Amelioration of memory impairment	
Shaoyao Gancao Tang	—	3×Tg-AD mice	0.4% in drinking water for 14 weeks	Reduced NLRP3, Aβ, and Tau	Chiu et al. (2021)
				Improved working and spatial memory	
Bushen-Yizhi	—	SAMP8 mice	1.46, 2.92, and 5.84 g/kg/day, p.o. for 30 days	Reduced ASC, NLRP3, iNOS, IL-1β, IL-18, and caspase-1	Hou et al. (2018)
				Attenuation of cognitive impairment	
Jiedu-Yizhi	—	Hippocampal CA1 Aβ25-35 injected rats	3.6, 7.2, and 14.4 g/kg/day, p.o. for 8 weeks	Decreased Aβ deposition, NLRP3, caspase-1, IL-1β, and IL-18	Wang et al. (2022)
				Rescue cognitive function	
Extracts of natural medicines					
*Ginkgo biloba* extract	—	TgCRND8 APP mice	Diet with 600 mg/kg (0.6%) for 5 months	Cognitive function improved	Liu et al. (2015)
				TNF-α and IL-1β decreased; caspase-1 activity and NLRP3 decreased	
Epimedii Folium and Curculiginis	—	Dorsal hippocampus Aβ1-42 injected rats	2 and 6 g/kg/day, p.o. for 30 days	TNF-α, IL-1β, and IL-6 decreased	Lan et al. (2017)
Rhizoma				MyD88, NLRP3 inflammasome, and cathepsin B decreased	
				Cognitive function improved	
Lychee seed polyphenol	—	Aβ1-42 induced BV2 cells	50, 100, 200 mg/kg/day, p.o. for 60 days	NLRP3, ASC, cleaved caspase-1, and IL-1β decreased	Qiu et al. (2020)
		APP/PS1 mice		Cognitive function improved	
Lychee seed polyphenol	—	APP/PS1 mice	175, 350, 700 mg/kg/day, p.o. for 5 weeks	Cognitive function improved	Xiong et al. (2021)
				NLRP3 inflammasome inactivation	
Virgin coconut oil	—	Icv injection of Aβ1-40 in Wistar rats	Diet with 8% and 10% VCO for 8 weeks	IL-1β, caspase-1, and NLRP3 decreased	Mirzaei et al. (2018)
				Spatial memory and learning ability improved	
Oleocanthal (OC) and extra-virgin olive oil (EVOO)	—	TgSwDI mice	Diet with 0.714 g/kg/day EVOO for 3 months	Improved BBB tightness	Al Rihani et al. (2019)
				Reduction of Aβ load, plaques, p-tau, IL-1β, and oxidative stress	
				NLRP3 and caspase-1 decreased	
				Memory and learning ability improved	
Oleuropein-rich olive leaf extract	—	5×FAD mice	Diet with 695 μg/kg/day for 3 months	Inhibition of NLRP3 activation	Abdallah et al. (2022)
				Reduced Aβ levels, enhanced BBB integrity and function	
*Picrorhiza kurroa*	—	5×FAD mice	200 mg/kg/day, p.o. for 8 weeks	Memory function improved	Kim et al. (2020)
				IL-1β, procaspase-1, NLRP3 decreased	
*Hericium erinaceus*	—	AlCl_3_ intraperitoneally injected rats	200 mg/kg/day, i.p. for 6 weeks	Memory functions and learning improved	Cordaro et al. (2021)
				Reduction of Aβ, p-tau, NLRP3, caspase-1, IL-1β, and IL-18	
Single compounds					
Artemisinin	Terpenoid	APP/PS1 mice	40 mg/kg/day, i.p. for 30 days	NF-κB activity decreased	Shi et al. (2013)
				NALP3 and IL-1β decreased	
Ginkgolide B	Terpenoid	LPS-induced BV2 cell model	25, 50, or 100 mg/kg/day, p.o. for 21 days	Learning and memory behavioral improved	Shao et al. (2022)
		SAMP8 mice		M2 microglial polarization; caspase-1 and NLRP3 decreased	
Oridonin	Terpenoid	Icv injection of Aβ1-42 in mice	10 mg/kg/day, i.p. for 15 days	Inhibits NLRP3 inflammasome	Wang et al. (2016)
				Learning and memory deficits improved	
Carnosic acid	Terpenoid	Aβ induced human iPSC-derived microglia	2 μM	Reduction of IL-1β	Satoh et al. (2022)
Dihydromyricetin	Flavonoid	APP/PS1	1 mg/kg/day, i.p. for 14 and 28 days	Inhibition of activated microglia	Feng et al. (2018)
				Reduction of caspase-1, IL-1β, NLRP3 inflammasome, and Aβ	
				Cognitive deficits amelioration	
Baicalin	Flavonoid	APP/PS1	103 mg/kg/day, p.o. for 33 days	Learning and memory deficits improved	Jin et al. (2019)
				IL-1β and IL18 decreased	
				NLRP3 inflammasomes and TLR4/NF-κB signaling inhibited	
Resveratrol	Flavonoid	Aβ1−42-induced BV2 cell model	Cotreatment of Aβ1−42 (20 μg/ml) and resveratrol (10 or 50 nM) for 24 h	TNF-α, IL-6, IL-1β, and cleaved caspase-1 decreased	Feng and Zhang (2019)
				TXNIP and NLRP3 protein expression decreased	
Quercetin	Flavonoid	SAMP8 mice	35 or 70 mg/kg/day, p.o. for 4 weeks	Cleaved-caspase 1, IL-1β, and IL-18 decreased	Li et al. (2021)
				Learning and memory improved	
Flavocoxid	Flavonoid	3×Tg-AD mice	20 mg/kg/day, i.p. for 3 months	Decreased Aβ deposition, NLRP3, IL-1β, and p-Tau	Bitto et al. (2017)
				Cognitive functions improved	
Liquiritigenin	Flavonoid	APP/PS1 mice	30 mg/kg/day, i.p. for 3 months	Reduction of NLRP3 and cleaved caspase-1	Du et al. (2021)
				Spatial learning and memory function improved	
Eriodictyol	Flavonoid	Hippocampal Aβ25-35 injected mice	10 mg/kg/day, p.o. for 4 weeks	NLRP3, caspase-1, ASC, IL-1β, IL-18, Aβ, and p-tau decreased	Guo et al. (2022)
				Improved memory and cognitive function	
Homoeriodictyol	Flavonoid	Hippocampal Aβ25-35 injected mice	10 mg/kg/day, p.o. for 4 weeks	NLRP3, caspase-1, ASC, IL-1β, IL-18, Aβ, and p-tau decreased	Guo et al. (2022)
				Improved memory and cognitive function	
Nobiletin	Flavonoid	APP/PS1 mice	Diet with 0.1% nobiletin for 15 months	Reduction of NLRP3 proteins	Wirianto et al. (2022)
				Inhibition of IL-1β and IL-18 mRNA	
				Improved memory function	
Thonningianin A	Flavonoid	APP/PS1 mice	0.25, 0.5, and 1.0 mg/kg, i.p. for 2 months	NLRP3, caspase-1, IL-1β, and Aβ decreased	Zhou et al. (2022)
				Learning and memory improved	
Pterostilbene	Polyphenol	Aβ1−42-induced BV2 cell model	Cotreatment of Aβ1−42 (5 μM) and pterostilbene (5 or 10 μM) for 24 h	TNF-α, IL-1β, and IL-6 decreased	Li et al. (2018)
				Reduction of caspase-1 and NLRP3	
Sulforaphane	Isothiocyanate	Aβ1–42-induced THP-1 macrophage	Cotreatment of Aβ1−42 (10 μM) and sulforaphane (5 μM) for 16 h	IL-1β decreased	An et al. (2016)
				NLRP3 protein expression decreased	
Astaxanthin	Carotenoid	APP/PS1 mice	Diet with 0.2% astaxanthin for 60 days	TNF-α, IL-1β, NLRP3, ASC, and caspase-1 decreased	Che et al. (2018)
				Learning and memory enhanced	

### 4.1 Traditional Chinese medicine formulations

Traditional Chinese medicine (TCM) has long been used in China and elsewhere. TCM formulas have been considered as potential therapeutic interventions for the prevention and treatment of AD due to their advantaged characteristics including multi-targeting, less toxic side effects, and multi-pathways. Currently, TCM formulas have been shown to be potent in modulating NLRP3 inflammasome activation by regulating its associated proteins and pathogens, such as ASC, caspase-1, IL-1β, ROS, NF-κB, and toll-like receptor 4.

A nutritional mixture (MBN) consisted of *cassia bark* (36.6%), *ginkgo leaf* (12.2%), *triphala* (12.2%), *turmeric root* (14.6%), and minor active ingredients including L-cysteine monohydrochloride and choline bitartrate has been evaluated on AD symptoms. Among these components, cassia bark, ginkgo, and turmeric were reported as having memory-ameliorating effects in AD mice and patients. MBN was orally administered to 5×FAD mice for 16 weeks at a concentration of 40 mg/kg/day; compared to the vehicle model, the Iba1+ activated microglia and GFAP+ astrocytes were significantly decreased in MBN-treated 5×FAD mice while memory decline was alleviated and caspase-1 activation was significantly inhibited (Kim et al., 2022). Shaoyao Gancao Tang (SG-Tang), a formulated Chinese herbal medicine, was made from *Paeonia lactiflora* and *Glycyrrhiza uralensis* at a 1:1 ratio, traditionally having been used for neuralgia, myospasm, and neuralgia. SG-Tang (0.4%) was added for 14 weeks to the drinking water of streptozotocin-induced 3×Tg-AD mice. The SG-Tang reduced the expression of NLRP1, NLRP3, Aβ, and Tau in the hippocampus and cortex, as well as improving spatial and working memories in Y maze and Morris water maze (Chiu et al., 2021). Bushen-Yizhi formula (BSYZ-F), consisting of *Cnidium monnieri* (L.) Cusson, *Paeonia suffruticosa* Andrews, *Panax quinquefolius* L, *Fallopia multiflora* (Thunb.) Harald, *Lycium Chinese* Mill, *Ligustrum lucidum* W. T. Aiton, in a proportion of 3:3:2:2:2:2, respectively, has been widely used for kidney deficiency. BSYZ-F alleviated cognitive impairment in SAMP8 mice at a dose of 1.46 g/kg for a 30-day treatment (Hou et al., 2018). Moreover, BSYZ-F inhibited NLRP3 inflammasome activation by downregulating inflammation-related proteins such as NLRP3, ASC, caspase-1, and IL-1β in MPTP-induced mice (Mo et al., 2018). Jiedu-Yizhi (JDYZF) formula is traditionally used as a tonic for the kidney and marrow, to resolve phlegm, and activate blood circulation and detoxification; it rescued the cognitive deficits in an Aβ25–35-induced rat model and reduced the expression of NLRP3, caspase-1, IL-1β, and IL-18 (Wang et al., 2022).

### 4.2 Extracts of medicinal plants

Medicinal plants have long been used against a variety of diseases in traditional medicines across the world. The phytochemical constituents present in medicinal plants play a paramount role in the treatment of inflammation. Therefore, we summarized current studies on medicinal extracts on NLRP3-mediated inflammation.

*Ginkgo biloba* is one of the oldest trees in the world, and its seeds are commonly used in traditional medicine. Its standardized leaf extracts have become a top-selling supplement in Europe and the United States. *G. biloba* leaf extract (EGb 761) was administered to TgCRND8 AD mice for 5 months at a dose of 600 mg/kg, and their cognitive function was remarkably improved in the Barnes Maze test; microglial inflammatory activation was inhibited, as well as the expression of TNF-α, IL-1β, and NLRP3, as well as caspase-1 activity (Liu et al., 2015).

Lychees are rich in polyphenols and are beneficial for spleen deficiency. Research indicates that lychee seed polyphenol improves tight junction protein expression by inhibiting NLRP3 inflammasome by activating AMPK/mTOR/ULK1-mediated autophagy in Aβ (25–35)-induced bEnd3 cells and APP/PS1 transgenic mice (Xiong et al., 2021). Another study demonstrated that lychee seed polyphenols suppress NLRP3 inflammasome by inhibiting the expression of NLRP3, ASC, cleaved caspase-1, and IL-1β secretion in Aβ1-42-stimulated BV-2 cells. Furthermore, lychee seed polyphenol improved the cognitive function and inhibited the NLRP3 inflammasome in APP/PS1 mice (Qiu et al., 2020).

Virgin coconut oil (VCO) has a variety of effects, including anti-oxidant and anti-inflammatory; thus, it is likely to be effective for AD treatment. An 8-week VCO diet downregulated the mRNA expression of IL-1β, caspase-1, and NLRP3; Aβ plaques and phosphorylated Tau were also reduced, and it improved memory and learning ability (Mirzaei et al., 2018). Oleocanthal (OC) and extra-virgin olive oil (EVOO) restored the BBB function and reduced Aβ load, plaques, and phosphorylated tau by inhibiting IL-1β and NLRP3 inflammasome; neurosynaptic function and learning and memory ability were also restored (Al Rihani et al., 2019). Additionally, when an oleuropein-rich olive leaf extract (OLE) diet (695 µg/kg/day) was administered to 5xFAD mice for 3 months, NLRP3 inflammasome activation was dramatically inhibited *via* NF-κB and RAGE/HMGB1 pathways. Aβ levels also decreased and BBB function recovered (Abdallah et al., 2022).

*Epimedii Folium* and *Curculiginis Rhizoma* are often prepared together in TCM to treat aging. Water extracts of these (1:1) were orally administered at doses of 2 and 6 g/kg/day for 30 days to dorsal hippocampus Aβ1-42-injected mice. Their spatial memory function and the activation of NLRP3 inflammasome were significantly ameliorated (Lan et al., 2017). *Picrorhiza kurroa*, a well-known herb in the Ayurvedic system of medicine, exhibits strong anti-inflammatory and nephroprotective effects. *P. kurroa* water extracts ameliorated memory impairment in 5xFAD mice, inhibited NLRP3 inflammasome activity, thus eased microglial neuroinflammation (Kim et al., 2020).

*Hericium erinaceus*, an edible fungus rich in β-glucan polysaccharides, has a long history of usage in TCM. *H. erinaceus* administration significantly ameliorates AlCl_3_-induced memory and learning deficits, and hippocampal neuronal degeneration. Phosphorylated Tau, Aβ, NLRP3, IL-1β, and IL-18 were also significantly reduced (Cordaro et al., 2021).

### 4.2 Natural compounds

#### 4.2.1 Terpenoids

Artemisinin, a well-known antimalarial sesquiterpene lactone, is isolated from the plant *Artemisia annua*. After daily intraperitoneal injection of artemisinin for 30 days with 40 mg/kg in 5-month-old APPswe/PS1dE9 transgenic mice, neuritic plaque was significantly decreased, and NF-κB activity and NALP3 inflammasome activation were dramatically inhibited (Shi et al., 2013).

Ginkgolide B is one of the main bioactive chemical compounds of *G. biloba*. In a recent study, it was indicated that ginkgolide B was able to convert M1 to M2 phenotype in LPS-induced BV2 microglia, as well as the cytokines IL-1β, IL-6, and TNF-α. In addition, Ginkgolide B inhibits NLRP3 inflammasome signaling in BV2 cells and in SAMP8 mice, and also attenuated learning and memory behavioral deficits (Shao et al., 2022).

Oridonin, an active diterpenoid isolated from the traditional Chinese herb *Rabdosia rubescens*, exerts diverse pharmaceutical and biological functions, especially anti-inflammatory effects. Oridonin is a specific and covalent inhibitor of NLRP3 inflammasome by forming a covalent bond with the cysteine 279 of NLRP3 in the NACHT domain to block the interaction between NLRP3 and NEK7, thereby inhibiting the activation of NLRP3 inflammasome (He et al., 2018). In addition, oridonin could prevent synaptic loss and improve behavioral symptoms in Aβ1–42-induced AD mice (Wang et al., 2016).

Carnosic acid, an abietane-type phenolic diterpene, is especially present in rosemary and has been reported as having multiple effects, including on neuroinflammation. It was reported that carnosic acid (2 µM) significantly ameliorated oligomeric Aβ-primed IL-1β release from human iPSC-derived microglia, and it blocks the inflammatory loop between microglia and neurons by inactivating NLRP3 inflammasome (Satoh et al., 2022).

#### 4.2.2 Flavonoids

Flavonoids, a group of natural compounds with variable phenolic structures, are derived from vegetables, grains, fruits, medicinal plants, tea, and wine. Flavonoids are well known for their beneficial effects on health attributed to their anti-inflammatory, anti-oxidative, anti-mutagenic, anti-cancer, and anti-alcohol intoxicative properties.

Dihydromyricetin is a flavonoid molecule derived from *Ampelopsis grossedentata* and has been demonstrated to exert anti-cancer, anti-oxidative, and anti-inflammatory effects. APP/PS1 mice was treated with dihydromyricetin for 2 or 4 weeks: their memory and cognitive deficits were significantly ameliorated, and the activated microglia and NLRP3 inflammasome were reduced in their hippocampus and cortex (Feng et al., 2018). *Scutellaria baicalensis* is a traditional medicinal plant in China that has long been used to treat various inflammatory diseases; its main pharmacological component is baicalin. This was orally administered at a dose of 103 mg/kg/day for 33 days to APP/PS1 mice: TLR4/NF-κB signaling mediated NLRP3 inflammasome activation was inhibited, and spatial memory dysfunction was attenuated (Jin et al., 2019). Resveratrol is well known as protecting against neuroinflammation and can protect microglia from Aβ1−42-induced inflammation by inhibiting the TXNIP/TRX/NLRP3 signaling pathway, leading to decreased NLRP3, caspase-1, and IL-1β (Feng and Zhang, 2019). Quercetin, another famous flavonoid, widely exists in vegetables and fruits. A daily supplement of quercetin has improved spatial learning and memory impairment in SAMP8 aging mice; furthermore, inflammatory factors such as cleaved-caspase 1, IL-1β, and IL-18 were downregulated after quercetin treatment (Li et al., 2021). Flavocoxid is a mixture containing baicalin and catechin and acts as a dual inhibitor of cyclooxygenase-2 and 5-lipoxygenase. Flavocoxid treatment reduced learning and memory loss, Aβ1-42, p-tau, and NLRP3 inflammasome in 3 × Tg-AD mice (Bitto et al., 2017). Liquiritigenin, a dihydroflavone monomer compound extracted from natural plant licorice, could significantly attenuate neuronal apoptosis in APP/PS1 transgenic mice. Meanwhile, liquiritigenin was able to convert M1-type microglia to M2 in both Aβ-induced BV2 cells and AD mice and alleviate the inflammation response by reducing NLRP3 and cleaved caspase-1, thus improving spatial learning and memory function (Du et al., 2021). Eriodictyol and homoeriodictyol are two dihydroflavonoids that exist widely in plants. Reports have demonstrated that these two molecules can penetrate the blood–brain barrier and ameliorate Aβ25–35-induced memory impairment in AD mice; they also inhibited NLRP3 inflammasome activation and ameliorated immune cell disorder (Guo et al., 2022). Nobiletin is a naturally occurring polymethoxylated flavonoid primarily that exists in citrus peel. A dietary supplement with nobiletin has potentially anti-inflammatory, anti-tumor, and cardioprotective properties. In a diet with 0.1% nobiletin administered for 15 months to APP/PS1 transgenic mice, the Aβ burden was significantly ameliorated as well as memory deficit. In addition, NLRP3 protein levels and the mRNA expression of IL1β/IL18 were dramatically decreased in their cortex after nobiletin treatment (Wirianto et al., 2022).

Thonningianin A, an ellagitannin flavonoid isolated from *Penthorum chinense* Pursh, was reported to induce autophagy in microglia mainly *via* the AMPK/ULK1 and Raf/MEK/ERK signaling pathways to degrade NLRP3 inflammasome. It also improved cognitive function, ameliorated the Aβ pathology, and inhibited NLRP3 inflammasome in APP/PS1 AD model mice (Zhou et al., 2022).

#### 4.2.3 Others

Pterostilbene, a natural dimethylated analog of resveratrol, inhibited the induction of NO and iNOS expression *via* stimulation with Aβ1−42 in BV2 microglia. Aβ1−42 activated NLRP3/caspase-1 inflammasome was inactivated by pterostilbene treatment (Li et al., 2018).

Isothiocyanate sulforaphane, derived from cruciferous vegetables, ameliorated the cognitive function of the Aβ-induced AD acute mouse model and decreased IL-1β production and NLRP3 protein expression (An et al., 2016).

A diet with 0.2% astaxanthin fed to APP/PS1 mice for 60 days enhanced learning and memory in the Morris water maze test and reduced Aβ plaques, hyperphosphorylation of tau, microglial activation, and NLRP3 inflammasome assembly (Che et al., 2018).

In summary, naturally derived constituents, especially terpenoids and flavonoids, are promising NLRP3 inhibitors for AD intervention.

## 5 Conclusion and perspectives

Memory loss is the key symptom of AD. Until now, there has been no treatment that can reverse memory deficit or even prevent worsening memory impairment. Over the past two decades, researchers have made great efforts to deconstruct the inflammasomes and reveal their role in disease progression. The NLRP3 inflammasome senses exogeneous stimuli, such as bacteria and viruses, and the endogenous signals that trigger the formation of caspase-1 in microglia and promote the generation and secretion of inflammatory cytokines, including IL-1β and IL-18. NLRP3 inflammasome activation is closely associated with Aβ load and Aβ-mediated tau pathologies. In addition, ER stress, Txnip, and Sharpin are also correlated with AD pathogenesis. Therefore, further investigations of the role of NLRP3 inflammasome in AD and its potential mechanisms may supply new therapeutic strategies for AD intervention.

TCM has been extensively applied in the prevention and treatment of AD both in a murine AD model and clinical therapies. A multitude of TCM formulations, extracts, and natural products have exhibited beneficial effects on the cognitive function of AD by regulating NLRP3 inflammasomes. We discovered from the literature that flavonoids and terpenoids are the main NLRP3 inhibitors in AD treatment. Whether other types of molecules exert NLRP3 inflammasome-inhibitive effects needs further investigation. Currently, machine learning-based virtual screening has been used in the discovery of preferred NLRP3 inhibitors. With the aid of the ZINC20 database, virtual screening has been performed by targeting the Walker A site and NACHT domain of NLRP3; two sets of predicted inhibitors, including steroid derivatives and indole rings contained molecules, were discovered (El-Sayed et al., 2022). Nevertheless, most naturally derived molecules have common shortcomings when they are applied in clinical studies; these include poor bioavailability (resulting from low water solubility, poor oral absorption, and digest/liver enzyme-induced rapid biotransformation), non-targeted distribution, and multiple pharmacokinetic patterns when incorporated into various forms of dosage. To overcome these limitations, chemical analogues can be designed and synthesized to inhibit rapid chemical degradation, as well as formulations of compounds in nanoparticles, liposomes, and phospholipid complexes to increase the targeting and effectiveness.

In clinical trials, some anti-inflammatory therapies, including non-steroidal anti-inflammatory drugs, failed to ameliorate cognitive deficits in AD patients. Owing to the complexity of AD, targeting multiple points in one or more pathways may be more efficacious than targeting a single node. In line with our hypothesis that optimizing the brain inflammatory and toxic microenvironment by regulating microglia polarization and promoting neurite regeneration may be a potential therapeutic strategy for AD, the multi-targets, multi-pathways, and less toxic side effects of TCM and TCM-derived compounds may provide new directions in the treatment of AD.
